# A cell-based large-scale screening of natural compounds for inhibitors of SARS-CoV-2

**DOI:** 10.1038/s41392-020-00343-z

**Published:** 2020-10-03

**Authors:** Zhe-Rui Zhang, Ya-Nan Zhang, Xiao-Dan Li, Hong-Qing Zhang, Shu-Qi Xiao, Fei Deng, Zhi-Ming Yuan, Han-Qing Ye, Bo Zhang

**Affiliations:** 1 Key Laboratory of Special Pathogens and Biosafety, Wuhan Institute of Virology, Center for Biosafety Mega-Science, Chinese Academy of Sciences, 430071 Wuhan, China; 2 University of Chinese Academy of Sciences, 100049 Beijing, China; 3 Hunan Normal University, School of Medicine, 410081 Changsha, China; 4 State Key Laboratory of Virology and National Virus Resource Center, Wuhan Institute of Virology, Chinese Academy of Sciences, 430071 Wuhan, Hubei China

**Keywords:** Drug screening, Microbiology

**Dear Editor,**

Coronavirus disease 2019 (COVID-19), caused by severe acute respiratory syndrome coronavirus 2 (SARS-CoV-2), has spread rapidly and developed into a global pandemic since its outbreak in December 2019. Currently, there is no antiviral treatment available for human use. Numerous compounds, such as remdesivir and chloroquine, have been reported to inhibit SARS-CoV-2 replication effectively in vitro, but for most of them, the in vivo efficacies against SARS-CoV-2 are still under clinical studies, and for chloroquine, a drug with prominent in vitro antiviral activity, it has been found no beneficial effect for COVID-19 patients in the recent largest study. It is thus urgent to speed up large-scale screening to discover drug candidates to treat COVID-19.

Recently, several high throughput screening (HTS) assays had been developed for SARS-CoV-2 antiviral discovery. A virtual screening and a fluorogenic protease enzymatic assay based on the main protease of SARS-CoV-2 have been established to screen the protease inhibitors. A reporter gene system had been developed to screen inhibitors targeting the −1 ribosomal frameshifting of SARS-CoV-2.^1^ These systems select the inhibitors targeting to one specific step during infection. Here, we established a cytopathic effect (CPE)-based HTS assay in Vero-E6 cells that are permissive to SARS-CoV-2 infection to screen for inhibitors aiming to the entire viral life cyle. The antiviral efficacy of compounds was determined by the reduction of CPE, which was quantified by measuring cell viability using CCK-8 assay. The HTS conditions, including the cell density, the multiplicity of infection (MOI) and the time of incubation were first optimized in a 96-well format. The final HTS conditions were at 5000 cells/well, 0.01 of MOI, 48 h of incubation to achieve maximum assay sensitivity (producing consistently > 90% CPE in the Vero-E6 cells at endpoint) for drug screening (Supplementary Fig. S1a and Fig. 1a).

**Fig. 1:**
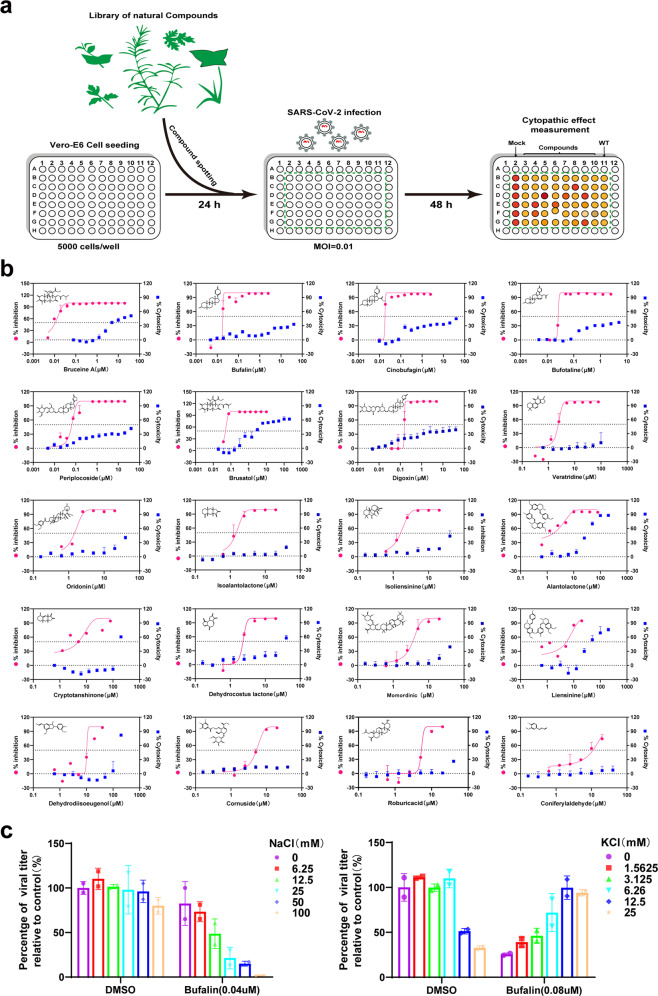
High throughput screening and identification of a natural compound library for inhibitors of SARS-CoV-2. **a** Flow chart of the cell-based HTS assay. Vero-E6 cells were seeded in 96-well plates one day prior to infection and infected with SARS-CoV-2 (MOI = 0.01) in the presence of tested compounds, and CPE induced by the virus was quantified by CCK-8 assay at 48 hpi. **b** Evaluation of anti- SARS-CoV-2 activity and cytotoxicity of the 17 newly discovered compounds and three previously reported CoVs inhibitors (bufalin, digoxin, and cryptotanshinone). At 24 hpi, the viral RNA levels in supernatants were measured by qRT-PCR assay. The cytotoxicity of the compounds at different concentrations was measured by a CCK-8 assay. The EC_50_ and CC_50_ were calculated by nonlinear regression analysis using GraphPad Prism 8.0 software. The selective indexes (SI) were calculated as the ratio of CC_50_ to EC_50_. **c** Addition of sodium and potassium assay. Vero-E6 cells seeded in 24-well plates were treated with DMSO or bufalin in the medium supplemented with NaCl (at a concentration of 0, 6.25, 12.5, 25, 50, or 100 mM) and KCl (at a concentration of 0, 1.5625, 3.125, 6.25, 12.5, or 25 mM) for 1 h, respectively, and then incubated with SARS-CoV-2 at an MOI of 0.01 for 24 h. The viral RNA levels in supernatants were determined by qRT-PCR assay. Inhibition rates were calculated as the percentage of infected cells normalized to DMSO-treated cells

Multiple known inhibitors, including remdesivir, chloroquine, neutralizing human antibody CB6^2^ and IFN-α were used as positive controls to validate the availability of the CPE-based HTS assay. Consistent with previous results, all these reagents provided protection against SARS-CoV-2 infection, and the Z′ values were 0.68, 0.56, 0.66, and 0.58, respectively (HTS assays with Z′ ≥ 0.5 are considered robust) (Supplementary Fig. S1b). These results demonstrated that this CPE-based HTS assay is reliable and robust for screening inhibitors of SARS-CoV-2.

As the compound is usually dissolved in DMSO, we also tested the effect of different concentrations of DMSO on SARS-CoV-2 replication to determine the optimal solvent concentration for the assay. As shown in supplementary Fig. S1c, DMSO had no effect on cell viability at ≤1% (v/v) concentration. Therefore, we chose 0.25% DMSO as the working concentration to dissolve the compounds.

To further verify the reliability of the selected conditions, we evaluated the inhibitory effects of different concentrations of chloroquine on SARS-CoV-2 infection under the above HTS condition. In parallel, viral NP protein expression and genomic RNA copies were detected by IFA and real-time PCR assay, respectively. In agreement with the results obtained from IFA and qRT-PCR assay, chloroquine showed a dose-dependent inhibition of CPE, and the resulting EC_50_ value of 2.65 μM was comparable to that determined by viral RNA copies (1.78 μM) (Supplementary Fig. S1d–f). These results further support the feasibility of the HTS assay for anti-SARS-CoV-2 drug screening. In addition, 10 µM of chloroquine was used as the positive control for the following HTS assay.

Under the established HTS assay condition, we screened a collection of natural compound library containing 1058 compounds to identify potential inhibitors of SARS-CoV-2 in cell culture (Fig. 1a). After the primary screening, 30 hits with > 50% protection from CPE were identified (Supplementary Fig. S2a). The Z′ values of each screening were between 0.5 and 1, with an average value of 0.7, which confirmed the reliability of our assays (Supplementary Fig. S2b). Among them, 12 drugs (bavachin, psoralidin, reserpine, tanshinone IIA, isobavachalcone, cryptotanshinone, lycorine, lycorine hydrochloride, fangchinoline, cepharanthine, tetrandrine, and bufalin) have been previously reported to be able to inhibit the infection of SARS-CoV, MERS-CoV, and other human coronaviruses; 4 of which (cepharanthine, lycorine, tetrandrine, and digoxin) were recently demonstrated to block the infection of SARS-CoV-2; 17 drugs are newly discovered inhibitors of SARS-CoV-2 in this study (Supplementary Fig. S2c).

We next evaluated the anti-SARS-CoV-2 effects of the 17 newly discovered compounds and 3 previously reported coronaviruses inhibitors (bufalin, digoxin, and cryptotanshinone) by measuring the alterations of viral genome levels when treatment with different concentrations of these hits. As shown in Fig. 1b, all tested compounds showed inhibitory effects on virus propagation in a dose-dependent manner with the EC_50_ values ranging from 0.011 to 11.03 µM. According to the results of CCK-8 assay, the CC_50_ values of these compounds were also calculated. 16 compounds showed the selective indexes (SI, defines as [CC_50_]/ [EC_50_]) > 10) (Supplementary Table S1), implying their potential as new antivirals against SARS-CoV-2 infection.

Through the literature review, we summarized the reported functions or targets of these 30 hit compounds. As listed in Supplementary Fig. S2c, a majority of these compounds target different signal pathways such as JAK/STAT, Nrf2, MAPK and NF-κB, implying the important roles of signal transductions during SARS-CoV-2 infection. Among the six hits targeting ion channels, four hits (bufalin, digoxin, cinobufagin, and bufotaline) are cardiac glycosides that inhibit the Na^+^/K^+^-ATPase. Notably, these cardiac glycosides displayed the most effective antiviral effects with nanomolar EC_50_ values (Fig. 1b).

Cardiac glycosides have been reported as potential broad-spectrum antiviral drugs.^3^ Na^+^/K^+^-ATPase is the only target of cardiac glycosides that has been found to date. It has reported the cardiac glycosides ouabain and bufalin could inhibit MERS-CoV infection by inhibiting the Na^+^/K^+^-ATPase mediated Src signaling without affecting its ion transport function.^4^ To confirm whether cardiac glycoside inhibits SARS-CoV-2 infection via blockade of Na^+^/K^+^-ATPase, we chose one cardiac glycoside, bufalin that had high selective index for further investigation. The cells were pre-treated with DMSO or bufalin in the presence of increasing concentrations of extracellular NaCl or KCl for 1 h prior to infection. The supernatants collected at 24 hpi were subjected to qRT-PCR for quantification of viral genomic RNA levels. The inhibitory effect of bufalin on SARS-CoV-2 was either enhanced by NaCl or suppressed by KCl in a dose-dependent manner (Fig. 1c). Such opposite effects observed in the NaCl and KCl cases confirmed that bufalin exerts the antiviral effect by targeting the ion transport function of Na^+^/K^+^-ATPase. Similar phenomena were found in digoxin, another kind of cardiac glycoside that inhibited the replication of zika virus and chikungunya virus,^5^ suggesting that such host proteins as Na^+^/K^+^-ATPase involved in the regulation of intracellular ion homeostasis may represent a broad-spectrum target for antiviral drugs. The Na^+^/K^+^-ATPase is involved in many cellular biosynthetic and signal pathways, the antiviral mechanism of cardiotonic steroids may also refer to multiple host factors. Further investigation of their cellular mechanism will help to identify the host factors and signal pathways involved in SARS-CoV-2 infection.

In summary, we have established a CPE-based HTS assay which are time-saving and allows for rapid screening of antivirals targeting the entire life cycle of SARS-CoV-2. Using this system, 1058 compounds from natural compound library were screened, and 30 hit drugs exhibiting good antiviral activities were identified, enriching the drug arsenal against SARS-CoV-2 infection.

## Supplementary information

Supplementary materials

## Data Availability

The data used and analyzed in this study are available in the main text and the Supplementary Materials. Any other raw data that support the findings of this study are available from the corresponding author upon reasonable request.
