# Supplementary material for: A cell-based large-scale screening of natural compounds for inhibitors of SARS-CoV-2
Source: Signal Transduct Target Ther. 2020 Oct 3;5:218. doi: 10.1038/s41392-020-00343-z (PMC7532339; doi:10.1038/s41392-020-00343-z)
Supplement: Supplementary file 1 — Supplementary materials [file 41392_2020_343_MOESM1_ESM.docx]

Supplementary Materials for

A cell-based large-scale screening of natural compounds for inhibitors of SARS-CoV-2

Zhe-Rui Zhang†, Ya-Nan Zhang†, Xiao-Dan Li†, Hong-Qing Zhang, Shu-Qi Xiao, Fei Deng, Zhi-Ming Yuan, Han-Qing Ye*, Bo Zhang*

Correspondence to: [*yehq@wh.iov.cn*](mailto:yehq@wh.iov.cn) or [*zhangbo@wh.iov.cn*](mailto:zhangbo@wh.iov.cn)

**This PDF file includes:**

Materials and Methods

Figures. S1 to S2

Tables S1

Materials and Methods

**Cell lines, viruses, antibodies and reagents**

Vero-E6 cells (ATCC number CRL-1686) were cultured at 37 °C with 5% CO_2_ in Dulbecco’s modified Eagle’s medium (DMEM; Invitrogen, Darmstadt, Germany) with 10% FBS, 100 U/ml penicillin and 100 mg/ml streptomycin. SARS-CoV-2 (WIV-04) was propagated in Vero-E6 cells and stored aliquots at −80℃ for experiments. The viral titer was determined by plaque assay. The rabbit anti-RP3-CoV NP protein antibody which is cross-reactive with the NP protein of SARS-CoV-2 was kindly provided by Prof. Zheng-Li, Shi (Wuhan institute of Virology, CAS). FITC-conjugated goat anti-mouse IgG was purchased from Protein Tech Group. The natural compound library was purchased from Weikeqi Biotech (Sichuan, China).

**Plaque assay**

The viral titer of SARS-CoV-2 was determined by single-layer plaque assay. The viral samples were serially diluted in 10-fold series, and each dilution was used to infect Vero-E6 cells (1×10^5^ cells per well) on 24-well plate for 1 h. After washing once with culture media, DMEM containing 2% FBS and 1% methylcellulose was added. After 3-4 days of incubation at 37 °C with 5% CO_2,_ these cells in plates were fixed with 3.7% formaldehyde and stained with 1% crystal violet in water. After washed with water, the number of plaques was recorded for viral titer quantification.

**qRT-PCR assay**

RNA was extracted from the supernatant of the infected cells using QIAamp viral RNA mini kit (52906, Qiagen) following the manufacturer’s protocol. qRT-PCR assay was performed using Luna® Universal Probe One-Step RT-PCR Kit (E3006). For genomic RNA quantification, the primer pairs (RBD-qF1: 5’- CAATGGTTTAACAGGCACAGG-3’, RBD-qR1: 5’- CTCAAGTGTCTGTGGATCACG-3’ and Probe: 5’-ACAGCATCAGTAGTGTCAGCAATGTCTC-3’) were used, and the number of genomic RNA copies was determined with a standard curve.

**HTS assay**

CPE-based HTS assay was developed in a 96-well plate format using chloroquine as a positive control and 0.25% DMSO as a negative control. The compounds from the library were all dissolved in DMSO at a stock concentration of 40 mM, and the working concentration is 10 µM. Vero-E6 cells were seeded into 96-well plates at the cell density of 5 × 10^3^ per well and cultured for 24 h at 37 °C, and then were infected with SARS-CoV-2 (MOI= 0.01). At 48 hpi, cell viability was examined using Cell counting Kit 8 (CCK-8) under the manufacturer’s instructions. Plates were read by SHE-3000 plate reader at 450 nm. The wells containing cells only, cells infected with virus, and virus-infected cells treated with chloroquine were used as internal controls. Percentage of cytopathic effect (CPE) inhibition was defined as [(test compound – virus control)/ (cell control – virus control)]×100. The “hit” compound was determined to be the compound that exhibited a %CPE inhibition of ≥ 50%. To evaluate the performance of the HTS assay, the Z’ factor values were calculated. The Z’ value was calculated as 1 – (3×standard deviation of cell control + 3×standard deviation of the virus control) /(mean cell control signal – mean virus control signal). The Z’ factor between 0.5 and 1 indicates an excellent assay with good separation between controls.

**Antiviral assay**

Vero-E6 cells were seeded in 24-well plates (8 × 10^4^ cells per well). The cells were incubated with different concentrations of compounds and infected with SARS-CoV-2 (MOI=0.01) at 37 °C for 24 h. Subsequently, the cell culture medium from each well was collected and subjected to qRT-PCR assay. The antiviral activity of compounds was expressed as 50% effective concentration (EC_50_) and was calculated by GraphPad Prism software 8.0.

**Cytotoxicity assay**

Vero-E6 cells were seeded in 96-well plates (1 × 10^4^ cells per well) and allowed to grow for 24 h before treatment. Then, serial 2-fold dilutions of compounds were added to the cells. At 48 h, the cells were incubated with 10 μL CCK-8 reagent (cell counting kit-8, Bimake) for 1 h at 37 °C. The absorbance at 450 nm was read by a Microplate Reader (Varioskan Flash, Thermo Fisher). Cell viability was expressed as a percentage of the treated cells to the control (untreated) cells. For each compound concentration, three wells were performed in parallel, and the mean values of the cell viability were calculated. The CC_50_ was calculated by nonlinear regression analysis using GraphPad Prism 8.0 software to determine the cytotoxic concentration at which 50% of the cells are viable.

**Addition of sodium and potassium assay**

Vero-E6 cells were incubated with DMSO or bufalin in the standard medium or the medium supplemented with increasing concentrations of NaCl (at a concentration of 0, 6.25, 12.5, 25, 50, or 100 mM) or KCl (at a concentration of 0, 1.5625, 3.125, 6.25, 12.5, or 25 mM). Then SARS-CoV-2 (MOI=0.01) was added in each well and incubated at 37℃ for 24 h, and the viral genomic RNA levels were measured by qRT-PCR assay. Results are presented as the percentages of infected cells normalized to DMSO-treated cells.

Figure. S1.


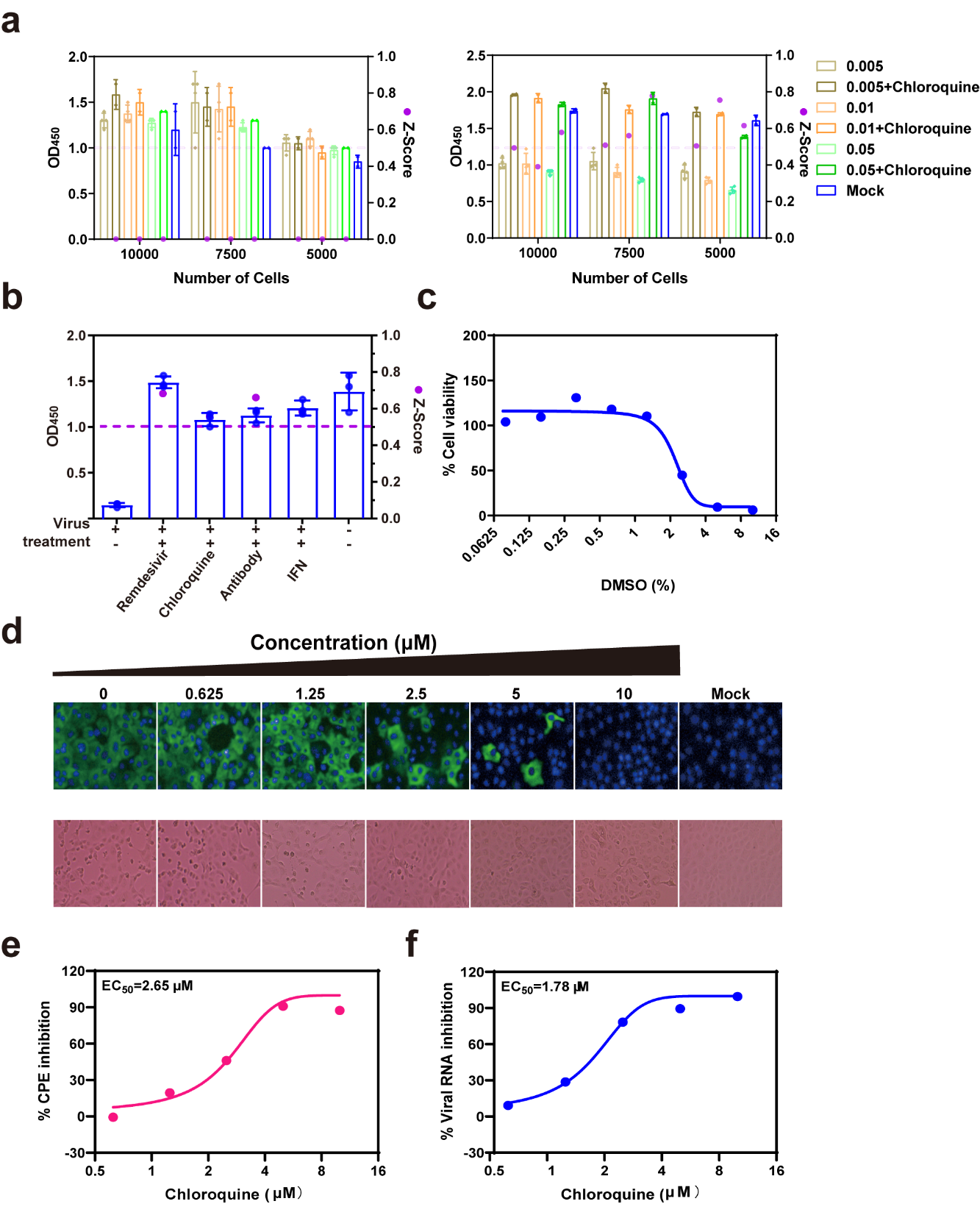


**Fig. S1. Establishment and Optimization of the cell-based HTS assay.** (a) Effects of different cell densities, multiplicities of infection and time courses of infection on the cell viabilities of the SARS-CoV-2 infected cells. Different numbers of Vero-E6 cells (5,000, 7,500 or 10,000) were seeded into each well of a 96-well plate, followed by infection with different MOIs of SARV-CoV-2 (0.005, 0.01 or 0.05) with or without treatment with the known inhibitor chloroquine. The cell viabilities were quantified at 24 h or 48 h after infection with the commercially available CCK8 kit. (b) Verification of the efficacy of CPE reduction assay for screening the compounds against SARS-CoV-2 replication in cell culture using known inhibitors which are remdesivir, chloroquine, neutralizing human antibody CB6 and IFN-α. (c-e) Inhibitory effects of different concentrations of chloroquine on SARS-CoV-2 replication by IFA analysis and CPE observation. Vero-E6 cells were infected with SARS-CoV-2 at an MOI of 0.01, and treated with different concentrations of chloroquine at the same time. After 48 hours infection, the CPE of each well was observed under a microscope. Then the cells were fixed and subjected to immunofluorescence assay using NP antibody and FITC-conjugated goat anti-rabbit IgG antibody as primary and secondary antibodies respectively. The cell images were captured under a fluorescence microscope (c). The cell viabilities were quantified by CCK8 assay (d) and the viral genome RNA levels were determined by qRT-PCR (e).

Figure. S2.


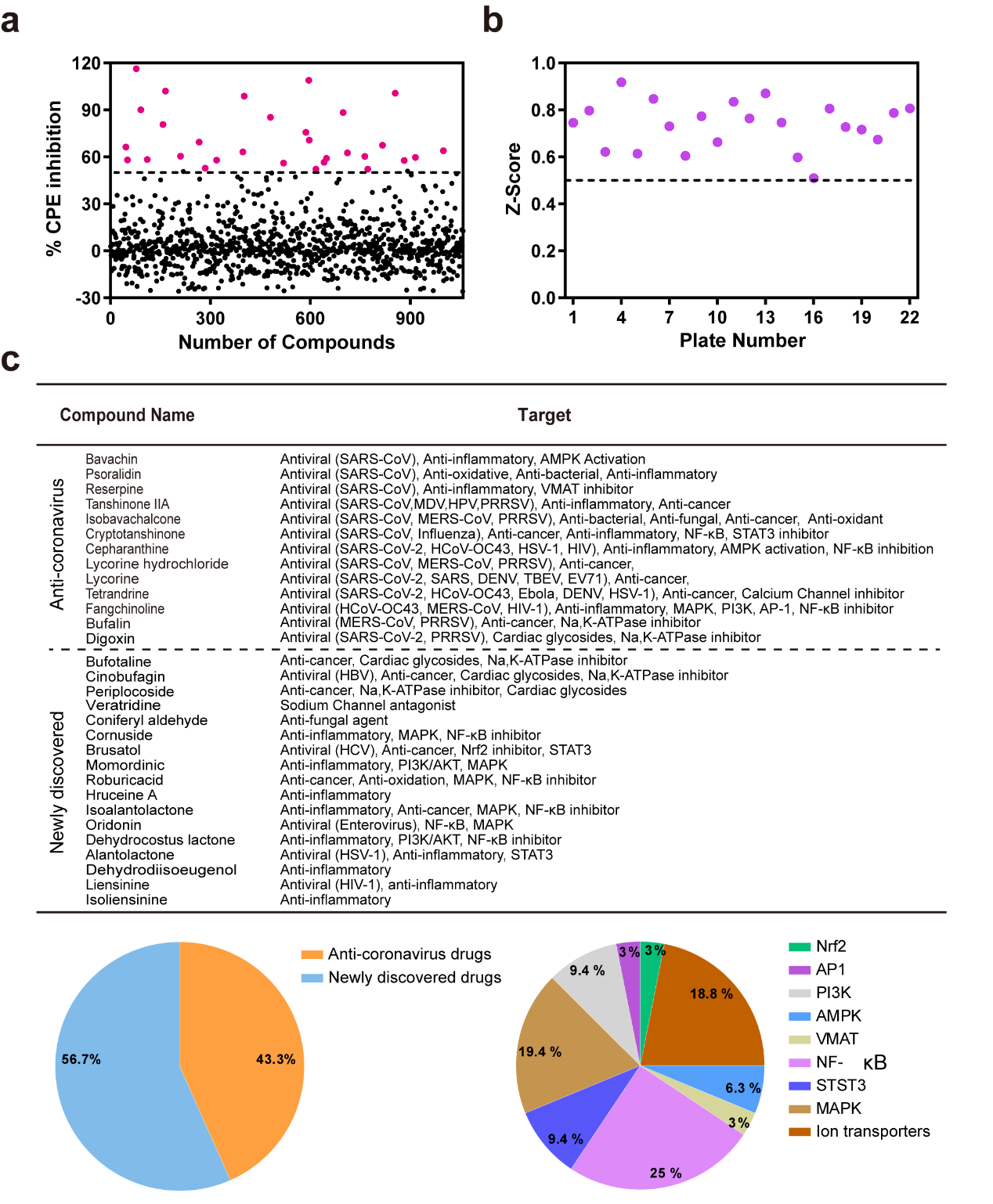


**Fig. S2. High-throughput screening of a natural compound library containing 1,058 drugs for inhibitors of SARS-CoV-2.** (a) Vero E6 cells were seeded in 96-well plates. After 24 hours, cells were infected with SARS-CoV-2 (MOI = 0.01) in the presence of tested compounds, and CPE induced by the virus was quantified by CCK8 assay at 48 hours post-infection. Scatter plot of HTS screened data of 1,058 drugs in the natural compound library. Hit drugs had greater than 50% CPE inhibition (represented by horizontal black dot line). (b) The Z’-value of each plate screening different compounds. (c) Summary and distribution of the known biological functions and targets of candidate compounds.

Table S1.

| **Hit compounds** | **Resources** | [**Structure**](javascript:;) | **EC_50_ (μM)** | **CC_50_ (μM)** | **SI** |
| --- | --- | --- | --- | --- | --- |
| Bruceine A | Brucea javanica |  | 0.011 | 31.4 | 2854 |
| Bufalin | Toad Venom |  | 0.018 | >40 | >2222 |
| Cinobufagin | Toad Venom |  | 0.018 | >40 | >2222 |
| Bufotaline | Toad Venom |  | 0.0259 | >40 | >1544 |
| Periplocoside | Toad Venom |  | 0.0657 | >40 | >608 |
| Brusatol | Brucea javanica |  | 0.0492 | 19 | 386 |
| Digoxin | Digitalis |  | 0.1541 | >40 | >260 |
| Veratridine | Lily family |  | 2.376 | >100 | >42.1 |
| Oridonin | Rabdosia |  | 1.462 | >40 | >27.4 |
| Isoalantolactone | Anula helenium |  | 1.483 | >40 | >27 |
| Isoliensinine | Nelumbo nucifera |  | 1.615 | 40 | 24.8 |
| Alantolactone | Anula helenium | 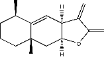 | 1.724 | 36.7 | 21.29 |
| Cryptotanshinone | Salvia miltiorrhiza |  | 5.024 | >100 | >19.9 |
| Dehydrocostus lactone | Saussurealappa |  | 2.322 | 36.2 | 15.59 |
| Momordinic | [Belvedere](javascript:;) [fruit](javascript:;) |  | 3.529 | >40 | 11.33 |
| Liensinine | Nelumbo nucifera |  | 2.537 | 25.4 | 10.01 |
| Dehydrodiisoeugenol | Aristolochia taliscana |  | 10.29 | >100 | 9.72 |
| Cornuside | Cornus officinalis |  | 5.262 | >40 | >7.6 |
| Roburicacid | Gentiana macrophylla |  | 5.267 | >40 | 7.59 |
| Coniferylaldehyde | eugenol |  | 11.03 | >40 | >3,64 |

**Table S1. EC_50_, CC_50_, SI values, resand structures of hit compounds.**
